# Azathioprine Increases the Risk of Non‐Melanoma Skin Cancer Among Organ Transplant Recipients; an Updated Systematic Review and Meta‐Analysis

**DOI:** 10.1002/cnr2.70473

**Published:** 2026-02-10

**Authors:** Amir Mohammad Salehi, Romina Rezaei, Alireza Sadeghi, Sanaz Omidi, Salman Khazaei, Bahareh Ebrahimi

**Affiliations:** ^1^ Fetal and Pediatric Cardiovascular Research Center, Children's Medical Center Tehran University of Medical Sciences Tehran Iran; ^2^ Student Research Committee Hamadan University of Medical Sciences Hamadan Iran; ^3^ Department of Epidemiology, School of Medicine Hamadan University of Medical Sciences Hamadan Iran; ^4^ Department of Dermatology, School of Medicine Hamadan University of Medical Sciences Hamadan Iran

**Keywords:** azathioprine, organ transplant, skin cancer, squamous cell carcinoma

## Abstract

**Background:**

Azathioprine (AZA) is a purine antimetabolite immunosuppressant that prevents the body from rejecting transplanted organs and is used in organ transplant recipients (OTRs). This study aimed to conduct a meta‐analysis to determine whether AZA usage has an increased risk of skin cancer in OTRs.

**Methods:**

To explore the association between AZA usage and skin cancer through observational studies we conducted a systematic search across PubMed, Scopus, and Web of Science databases. A random effects model, subgroup analysis, and heterogeneity assessment were used for meta‐analysis. The quality of the included studies was assessed using the Newcastle Ottawa scale checklist.

**Results:**

A total of 27 studies with 21 405 patients were included to the quantitative analysis. the overall summary estimate for non‐melanoma skin cancer (NMSC) risk in relation to AZA treatment according Odds ratio (OR) estimates, relative risk (RR) estimates and hazard ratio (HR) estimates were 1.83 (95% confidence interval (CI): 1.22, 2.75), 2.09 (CI: 1.41, 3.10), and 1.12 (CI: 0.93, 1.34), respectively. There was a substantial heterogeneity between studies in all types of OR estimates (I2 = 80.75%), RR estimates (I2 = 65.58%) and HR estimates (I2 = 54.63%). In the subgroup analysis, there was a significant increase in squamous cell carcinoma (SCC) risk in all three estimate effects while regarding basal cell carcinoma (BCC) none of them were significant.

**Conclusion:**

Our findings indicate that OTRs treated with AZA are at an increased risk for SCC and NMSC. Therefore, it is recommended to prioritize monitoring for skin cancer in OTRs treated with AZA.

**Abbreviations**
AZA: Azathioprine
BCC: Basal Cell Carcinoma
CI: Confidence Intervals
HR: Hazard Ratio
NMSC: Non‐Melanoma Skin Cancer
NOS: Newcastle‐Ottawa scale
OR: Odds Ratio
OTR: Organ Transplant Recipients
RR: Relative Risk
SCC: Squamous Cell Carcinoma
SIR: Standardized incidence ratio

## 1 Introduction

Immunosuppressants are vital treatments in organ transplant recipients (OTRs). Some commonly used agents include corticosteroids and nonsteroid immunosuppressants. The latter include azathioprine (AZA), mycophenolate mofetil, tacrolimus, and cyclophosphamide [1]. AZA, a purine antimetabolite immunosuppressant, was first used for clinical immunosuppression in 1961. It is a drug that prevents the body from rejecting a transplanted organ. AZA is typically used in combination with other drugs after a kidney transplant. It can also be used to treat severe rheumatoid arthritis and ulcerative colitis [2].

Skin cancer is the most common type of cancer in OTRs of Caucasian descent, and its incidence is steadily increasing worldwide [3]. Skin cancer is classified into two main categories: melanoma skin cancer and non‐melanoma skin cancer (NMSC) [4]. Non‐melanoma skin cancer is further classified into two types: squamous cell carcinoma (SCC) and basal cell carcinoma (BCC). SCC accounts for 25% and BCC for 70% of NMSCs [3].

BCC is less invasive than SCC, but both exhibit some degree of malignancy. If diagnosed early, they have a good prognosis [3]. Therefore, an early diagnosis and treatment of these skin lesions in AZA usage patients may improve post‐transplant outcomes [5].

To date, only two meta‐analyses have been conducted regarding the association between AZA and the risk of skin cancer in OTRs. They showed that AZA was a risk factor for SCC; however, no significant associations between AZA treatment and BCC or NMSC risk were observed [6]. Moreover, the previous review included studies only up to 2016 and did not cover the Web of Science database. Therefore, this study aimed to conduct an updated meta‐analysis to determine whether AZA usage is associated with an increased risk of skin cancer in OTRs. Unlike previous work, we incorporated studies published up to November 2025, included additional databases, analyzed OR, RR, and HR separately, and conducted multiple subgroup evaluations, providing novel clinical insights into the risk of non‐melanoma skin cancer in transplant recipients using azathioprine.

## 2 Materials and Methods

We performed the current systematic review and meta‐analysis according to the Preferred Reporting Items for Systematic Reviews and Meta‐Analyses (PRISMA) guideline [7]. Moreover, we registered the study protocol at the International Prospective Register of Systematic Reviews (PROSPERO) with register number CRD42023485231, Date: 11/21/2023.

### 2.1 Inclusion Criteria

We included primary studies that examined the risk of skin cancer development in OTR treated with AZA. Studies that mentioned the use of AZA in any treatment combination were considered, but those that did not provide a clear definition of the immunosuppressive regimens or had all comparison groups containing AZA were excluded. Additionally, studies that only examined the dosage of immunosuppressants were not included due to the lack of consistency in dosage assessment, which made it difficult to compare the results. Also, for inclusion in this meta‐analysis, studies needed to report odds ratios (OR), relative risks (RR), or hazard ratios (HR) estimates with 95% confidence intervals (CI). Studies lacking adequate data for outcome measurement were omitted. Furthermore, we excluded editorials, case reports, case series, systematic reviews, as well as in vitro and animal studies.

### 2.2 Information Sources and Search

PubMed (Medline), Web of Science, Scopus, and Science Direct were systematically queried up to November 30, 2025, using a combination of keywords, including (Azathioprine OR 6‐thiopurine OR immunosuppressant* OR immunosuppressive agent) AND (skin cancer OR skin neoplas OR squamous cell cancer OR squamous cell carcinoma OR basal cell cancer OR basal cell carcinoma OR SCC OR BCC OR non‐melanoma skin cancer OR nonmelanoma skin cancer OR NMSC OR melanoma OR malignant melanoma) AND (transplant*). Additionally, reference lists were scrutinized to uncover additional relevant sources.

### 2.3 Study Selection

The Population, Exposure, Comparison, and Outcome (PECO) model was utilized to determine eligible studies. In this model, the Population comprised patients with a history of organ transplantation, the Exposure was treated with AZA, the Comparison involved non‐treated with AZA, and the Outcome focused on skin cancer. Two researchers (AS and RR) independently reviewed all titles and abstracts, with full texts of potentially eligible studies considered. Any discrepancies between the two authors were resolved through discussion.

### 2.4 Data Extraction

Information extracted from the included studies was inputted into Stata software. This included details such as the first author, year of article publication, study location, study design, population size, age at transplantation, organ type, transplant period, exposure, follow‐up time, outcome category.

### 2.5 Quality Assessment

We used the modified Newcastle‐Ottawa scale (NOS) to assess the quality of observational articles [8]. The NOS includes three items: participant selection, comparability of skin cancer rates between OTR treated with AZA and those without treated with AZA, and outcome assessment. The scale ranges from 0 to 9, with the lowest and highest quality scores being 0 and 9, respectively. Scores between 0 and 6 indicate low quality, while scores between 7 and 9 indicate high quality.

### 2.6 Statistical Analysis

The relative risk (RR), odds ratios (ORs), and hazard ratios (HRs) of studies were pooled for the meta‐analysis. Higgins I^2^ and Cochrane Q statistics were used to evaluate the degree of heterogeneity among the included studies. In case of significant and high heterogeneity (P‐value for Q < 0.05 and I2% > 50%), the random effect model was used for pooling of the effect sizes. Egger's and Begg's tests were used to assess potential publication bias in a meta‐analysis via funnel plot asymmetry. All statistical analyses were performed using STATA version 17.0 software (Stata Corporation, College Station, TX, USA).

### 2.7 Subgroup Analysis

Additional analysis was conducted to identify significant variations among subgroups and explore the reliability of the correlation between AZA and the risk of skin cancer in OTR. Six predetermined subgroups were examined, which include cancer type, study design, adjustment, drug regimen, study quality, and organ transplant type.

## 3 Result

**FIGURE 1:**
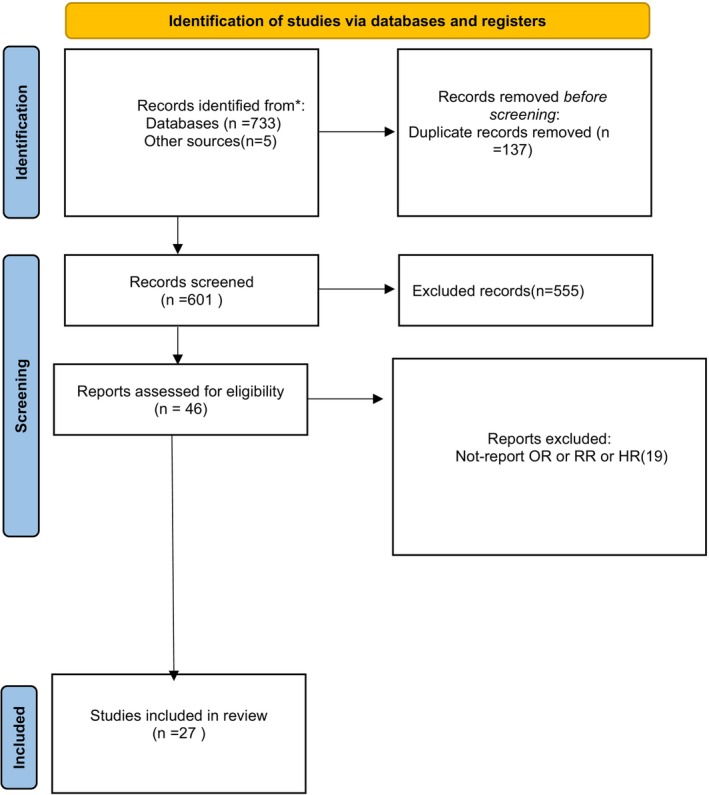
Prisma flowchart regarding study search and selection.

In November 2025, our search efforts yielded a total of 738 records. Following the elimination of duplicates, we conducted a thorough review of 601 papers. Subsequently, 555 articles were excluded from the meta‐analysis after initial screening and 19 potentially relevant articles were excluded after comprehensive examination of their full texts. Ultimately, our quantitative and meta‐analysis incorporated a total of 27 studies, as illustrated in (Figure 1).

Characteristics of 27 included studies to the meta‐analysis are presented in (Table 1). All of them were conducted in North and South America, European countries, and Oceania. 20 studies had cohort design, 6 had case–control, and one had cross‐sectional design. The study population size ranged from 116 to 3393. All of the information regarding age at transplantation, organ type, transplant period, exposure type, follow‐up time, and outcome category are shown in (Table 1).

**TABLE 1:** Characteristics of 27 included studies to the meta‐analysis.

First author	year	Study location	Study design	Population size	Male ratio	Age at transplantation	Organ type	Transplant period	Exposure (1)	Follow‐up time	Outcome category	Study quality
Bachelet [9]	2021	France	Case–control	202	49	mean Case: 48.4 Control: 50.5	Kidney	2005–2014	T/M/P T/A/P C/M/P mTor‐inhibitor/MP	Median: 43.3 months	SCC BCC	High
Bašić [10]	2022	Croatia	Cohort	1232	60	NM	Kidney	1974–2014	A	NM	SCC BCC	High
Brewer [11]	2009	United states	Cohort	312	73	mean: 47.4	Heart	1988–2006	A	Mean: 6.7 years	SCC BCC	High
Carroll [12]	2010	UK	Cohort	116	84	median with cancer: 47 without cancer: 46	Kidney	NM	A	Median: 340 days	SCC	Low
Coghil [13]	2016	United states	case–control	494	NM	48 to 62 years	Heart Kidney Kidney‐Pancreas	1998–2005	A	NM	SCC	High
Dusendang [14]	2022	United states	Cohort and nested case–control	3308	NM	NM	Kidney Lung Liver Heart Multiple	2009–2019	A	Median Case: 3.9 years Control: 4.2 years	SCC	High
Hamandi [15]	2018	United States, Canada, France, Germany, Italy, the Netherlands, Spain, Switzerland, and Australia	Cohort	900	53	NM	Lung Heart‐Lung	2005–2008	C/A T/A	Median: 3.51 years	SCC	High
Ingvar [16]	2010	Sweden	Case–control	396	68	median: 51	Kidney Liver Kidney‐Liver Kidney‐Pancreas Heart‐Lung	1970–1997	A A/P C/A/P	Median: 6.6 years	SCC	Low
Jensen [17]	1999	Norway	Cohort	2561	65	median K: 47 years H: 52	Kidney Heart	1963–1992	A/P C/A/P	median K: 4.8 years H: 3.5 years	SCC	High
Joly [18]	2010	France	Case–control	300	70	NM	Kidney Heart	NM	A	NM	SCC	Low
Sanders [19]	2015	United states	Case–control	388	61	mean Case: 63.2 Control: 52.6	Kidney Heart	Kidney (1970–2011) Heart (1985–2009)	A	NM	SCC	High
Mackenzie [20]	2010	New Zealand	Cohort	384	58	mean: 41.5	Kidney	1972–2007	P/C P/A P/A/C P/M/C P/M/T	Median: 5.3 years	NMSC	High
Molina [21]	2010	Spain	Cohort	3393	85	mean: 51.4	Heart	1984–2003	A	Median: 5.2 years	SCC BCC NMSC	Low
Vecchiato [22]	2021	Italy	Cohort	247	59	mean: 43	Lung	1995–2016	AP	Median: 2.9 years	SCC BCC	Low
Vos [23]	2018	Netherlands	Cohort	544	49	median: 49.1	Lung	1990–2016	A A/M	NM	SCC	High
Wisgerhof [24]	2010	Netherlands	Cohort	1906	62	median: 42	Kidney	1966–2006	A	Median: 20.6 years	SCC BCC	High
Bouwes Bavinck [25]	1996	Australia	Cohort	1098	57	mean with cancer: 46 without cancer: 41	Kidney	1969–1994	A before1980 A after 1980 C then Switch to A C/A	Median cancer: 8.9 years without cancer: 3.9 years	SCC BCC NMSC	High
Fuente [26]	2003	Spain	Cohort	174	68	median: 45	Kidney	1989–1999	C/A/P	Median: 6 years	NMSC	Low
Keller [27]	2010	Switzerland	Cohort	243	63	NM	Kidney	2002–2005	A	NM	SCC	Low
Navarro [28]	2008	Spain	Cohort	1017	NMSC patients: 71	NM	Kidney	1979–2007	A	Median: 10 years	NMSC	High
Ramsay [29]	2003	Australia	Cohort	361	60	median: 40.2	Kidney	NM	A/P C/A C/A/P	Median: 7.56 years	SCC BCC	High
Rashtak [30]	2015	United states	Cohort	166	NM	mean: 52	Lung Heart‐Lung Heart‐Lung‐Liver	1990–2011	A	Median: 3 years	SCC BCC any skin cancer	Low
Geusau [31]	2008	Austria	Cohort	322	81	54.02	Heart	1984–2003	C/A/P	Median: 74.18 months	NMSC	High
Vieira [32]	2023	Brazil	Cross‐sectional	308	58	48	Kidney	2015–2020	A	—	NMSC	Moderate
Marro [33]	2025	Italy	Cohort	568 Case: 42 Control:42	Case: 79 Control: 79	Median: 52	Heart	1990–2024	T/A	Median: 18.75 years	SCC Any skin cancer	High
Pinho [34]	2016	Portugal	Case–control	288	66	Median: 47	Kidney	2000–2009	A C/A/P	Median: 3.67 years	NMSC	Moderate
Zhu [35]	2025	New Zealand	Cohort	177	60	Mean: 47	Kidney	2004–2015	A	Median: 9 years	NMSC	High

Abbreviations: A, Azathioprine; C, Cyclosporine; M, Mycophenolate mofetil; NM, not mentioned; P, Prednisone; T, Tacrolimus.

**FIGURE 2:**
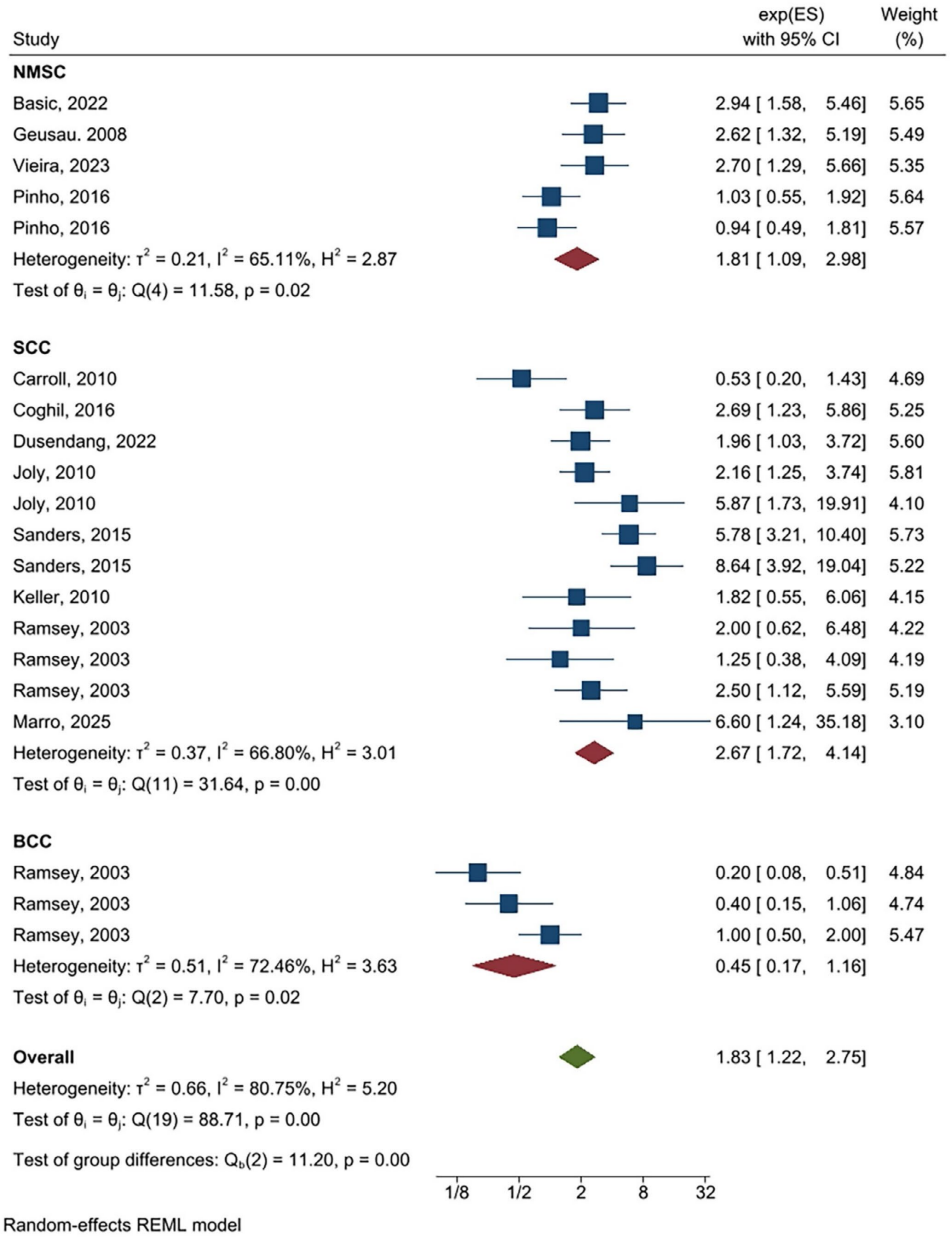
Random‐effects meta‐analysis of association of azathioprine use with non‐melanoma skin cancers (pooled ORs).

**FIGURE 3:**
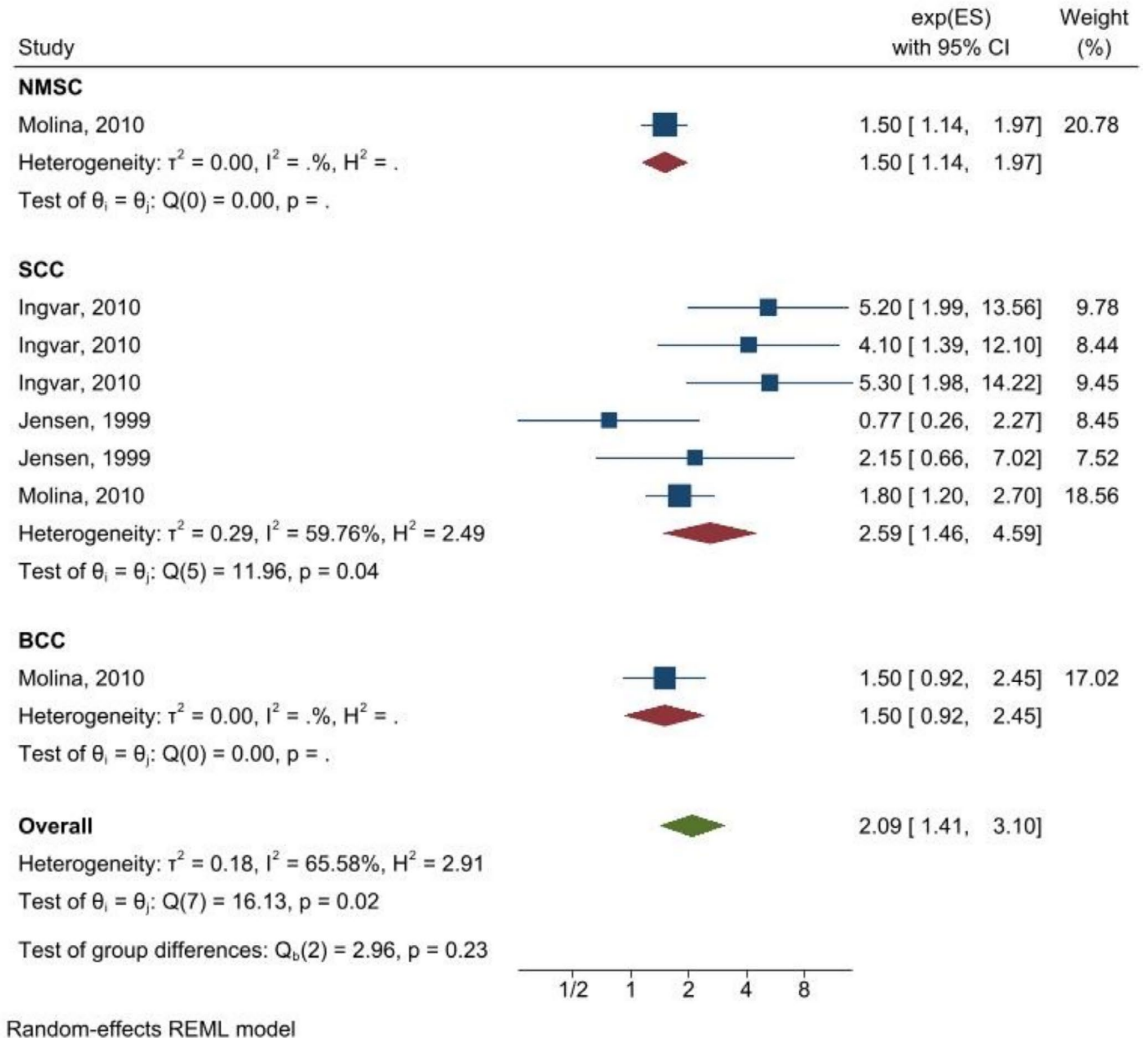
Random‐effects meta‐analysis of association of azathioprine use with non‐melanoma skin cancers (pooled RRs).

**FIGURE 4:**
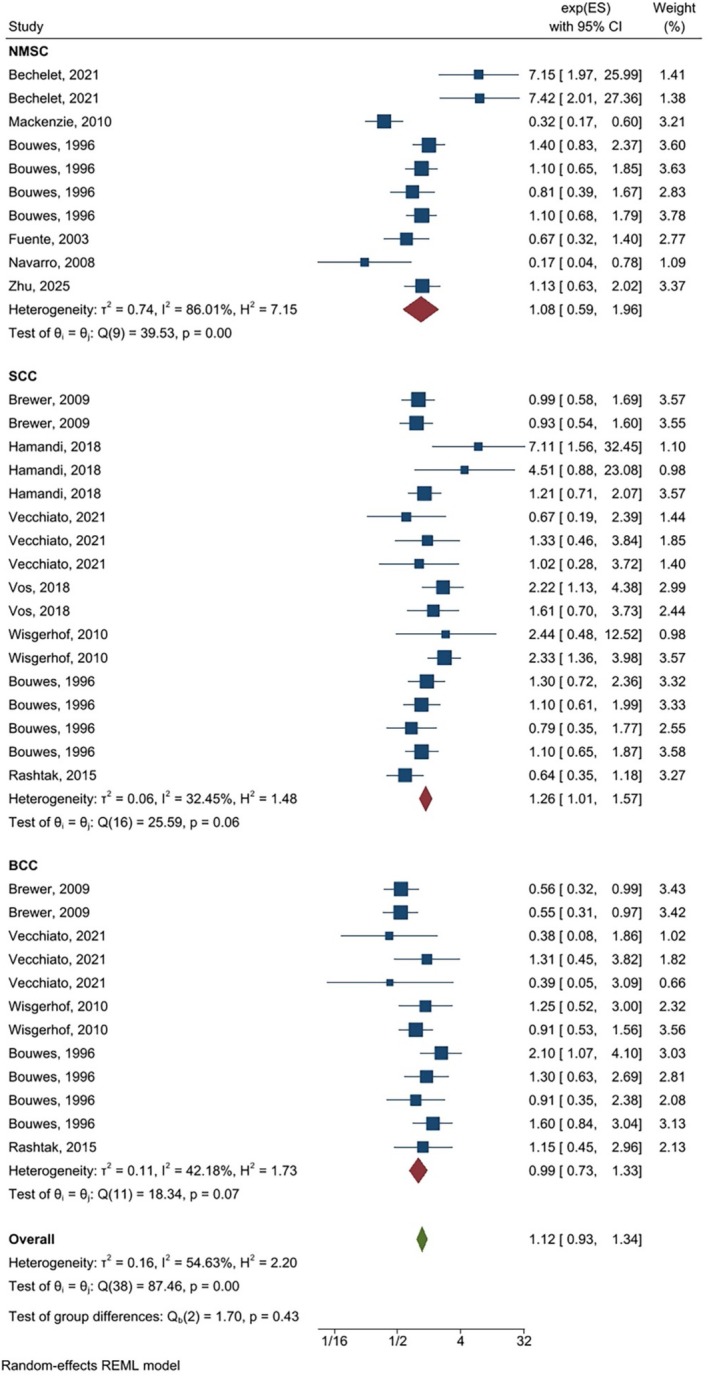
Random‐effects meta‐analysis of the association of azathioprine use with non‐melanoma skin cancers (pooled HRs).

As shown in (Figures 2, 3, 4) the overall summary estimate for NMSC risk in relation to AZA treatment according to OR estimates, RR estimates, and HR estimates was 1.83 (1.22, 2.75), 2.09 (1.41, 3.10), and 1.12 (0.93, 1.34), respectively. There was substantial heterogeneity between studies in all types of OR estimates (I2 = 80.75%), RR estimates (I2 = 65.58%), and HR estimates (I2 = 54.63%).

Sub‐group analysis of association of AZA use with NMSC by cancer type, study design, adjustment, drug regimen, study quality and organ type is presented in (Table 2).

**TABLE 2:** Sub‐group analysis of azathioprine use by cancer type, study design, adjustment, drug regimen, study quality and organ type.

		Number of studies	Odds ratio		Number of studies	Hazard ratio		Number of studies	Relative risk	
			OR (95% CI)	I2		HR (95% CI)	I2		RR (95% CI)	I2
Cancer type	NMSC	5	**1.81 (1.09, 2.98)**	65.11%	10	1.08 (0.59, 1.96)	86.01%	1	**1.50 (1.14, 1.97)**	_
	SCC	12	**2.67 (1.72, 4.14)**	66.80%	17	**1.26 (1.01, 1.57)**	32.45%	6	**2.59 (1.46, 4.59)**	59.76%
	BCC	3	0.45 (0.17, 1.16)	72.46%	12	0.99 (0.73, 1.33)	42.18%	1	1.50 (0.92, 2.45)	_
Study design	Case–control	8	**2.63 (1.50, 4.64)**	81.32%	2	**7.28 (2.91, 18.23)**	0.00%	3	**1.55 (1.27, 1.89)**	0.00%
	Cohort	11	1.29 (0.72, 2.30)	77.06%	37	1.06 (0.90, 1.25)	48.32%	5	**4.89 (2.74, 8.73)**	0.00%
	Cross‐sectional	1	**2.70 (1.29, 5.66)**	_	_	_	_	_	_	_
Adjustment	Crude	9	**2.05 (1.25, 3.37)**	77.26%	19	1.18 (0.89, 1.58)	57.06%	6	**2.34 (1.48, 3.71)**	74.13%
	Adjusted	11	1.65 (0.87, 3.13)	82%	20	1.07 (0.85, 1.35)	54.87%	2	1.25 (0.46, 3.41)	36.60%
Drug regimen	Single	11	**2.53 (1.62, 3.97)**	75.27%	26	1.16 (0.95, 1.41)	51.95%	4	**1.66 (1.36, 2.03)**	0.00%
	Dual	5	0.95 (0.30, 3.02)	80.34%	11	1.22 (0.94, 1.59)	0.00%	2	1.78 (0.34, 9.15)	78.21%
	Triple	4	1.55 (0.89, 2.69)	59.29%	2	**0.45 (0.22, 0.93)**	55.59%	2	**3.59 (1.49, 8.62)**	24.11%
Study quality	High	14	**1.96 (1.15, 3.33)**	82.47%	30	1.20 (0.97, 1.48)	63.53%	2	1.25 (0.46, 3.41)	36.06%
	Low	6	1.55 (0.0.85, 2.79)	73.51%	9	0.79 (0.57.1.10)	0.00%	6	**2.34 (1.48, 3.71)**	74.13%
Organ type	Kidney	_	_	_	22	1.18 (0.93, 1.50)	61.47%	_	_	_
	Lung	_	_	_	8	1.28 (0.86, 1.92)	10.89%	_	_	_
	Heart	_	_	_	4	0.74 (0.54, 1.01)	20.74%	_	_	_
All studies		20	**1.83 (1.22, 2.75)**	80.75%		1.12 (0.93, 1.34)	54.63%	8	**2.09 (1.41, 3.10)**	65.58%

*Note:* Statistically significant results (*P*‐value < 0.05) are formatted in bold.

The association between AZA use with NMSC according cancer type for SCC was significant for OR estimate [OR = 2.67, (95% CI: 1.72, 4.14)], HR estimate [HR = 1.26, (95% CI: 1.01, 1.57)], and RR estimate [RR = 2.59, (95% CI: 1.46, 4.59)], while regarding BCC none of them were significant. Results of the sub‐group analysis according to the study design indicated that in case–control studies all of the OR, HR, and RR estimates showed a significant association between azathioprine use and non‐melanoma skin cancer, but in cohort studies only RR showed a significant association [RR = 4.89, (95% CI: 2.74, 8.37)]. Moreover, after adjustment for confounding variables, the results of the crude estimate for OR and RR estimates were significant. Results of the sub‐group analysis for drug regimen for single drug, OR and RR estimates showed a significant association. None of them for dual drugs were significant and for triple drug only RR and HR estimates showed a significant association. Regarding organ type, pooling of the HR estimates could not show a significant association.

### 3.1 Publication Bias

**FIGURE 5:**
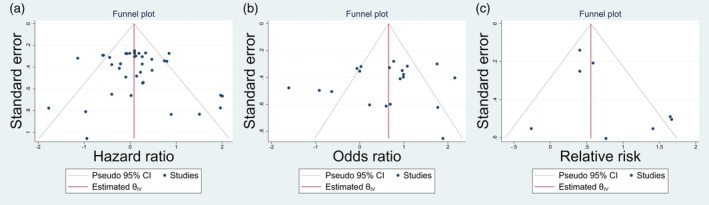
Funnel plot of the association between azathioprine use with non‐melanoma skin cancer by (a) OR estimate, (b) HR estimate and (c) RR estimate.

Begg and Egger's tests were applied to assess publication bias among studies. There was not significant evidence of publication bias among studies by Begg and Egger tests (*p* = 0.7212 and *p* = 0.9883, respectively) for OR estimate, and (*p* = 0.6457 and *p* = 0.2160, respectively) for HR estimate. Unlike Begg's test (*p* = 0.5362), Egger's test revealed significant publication bias for RR estimate (*p* = 0.0232). Moreover, the symmetric shape of the funnel plot also approves non‐evidence of publication bias (Figure 5).

The results of trim‐and‐fill analyses indicated potential small‐study effects in the RR and OR data, with two and one imputed missing studies respectively, leading to modest reductions in the pooled estimates after adjustment. No evidence of missing studies was found for HR estimates, suggesting minimal publication bias in this subset. Importantly, even after correction for potential publication bias, the association between azathioprine use and increased risk of non‐melanoma skin cancer remained statistically significant for RR and OR, supporting the robustness of our findings. These results and their corrections are summarized in the Table 3.

**TABLE 3:** Trim‐and‐Fill analysis results for publication bias assessment in Meta‐Analyses of RR, OR, and HR estimates.

Effect measure	Original pooled estimate (95% CI)	Number of imputed studies	Adjusted pooled estimate (95% CI)
Relative Risk (RR)	2.09 (1.41, 3.10)	2	1.82 (1.26, 2.63)
Odds Ratio (OR)	2.06 (1.56, 2.73)	1	1.93 (1.45, 2.56)
Hazard Ratio (HR)	1.12 (0.93, 1.35)	0	1.10 (0.91, 1.33)

### 3.2 Quality Assessment

According to the Newcastle‐Ottawa scores, most of the included studies were assessed as high quality. 18 studies were high quality and 9 studies were low quality.

### 3.3 Sensitivity Analysis

To evaluate the robustness of the pooled effect estimates for odds ratio (OR), hazard ratio (HR), and risk ratio (RR), leave‐one‐out sensitivity analyses were performed. In this procedure, each study was sequentially omitted, and the meta‐analysis was recalculated to determine whether any single study had a disproportionate impact on the overall effect size. The analyses showed that exclusion of any individual study resulted in minimal changes to the pooled estimates and their 95% confidence intervals (Table 4). This indicates that the results are stable and not unduly influenced by any single study, confirming the reliability of the pooled effect sizes across the included studies.

**TABLE 4:** Leave‐One‐Out Sensitivity Analysis for Pooled OR, HR, and RR.

Study omitted	OR (95% CI)	HR (95% CI)	RR (95% CI)
1	1.83 (1.24–2.69)	1.09 (0.91–1.30)	2.04 (1.43–2.90)
2	1.83 (1.24–2.69)	1.09 (0.91–1.30)	2.02 (1.41–2.90)
3	1.78 (1.18–2.68)	1.12 (0.93–1.34)	2.04 (1.43–2.90)
4	1.83 (1.24–2.69)	1.13 (0.93–1.36)	2.03 (1.42–2.91)
5	1.83 (1.24–2.69)	1.13 (0.93–1.36)	2.02 (1.43–2.89)
6	1.83 (1.24–2.69)	1.15 (0.95–1.38)	2.04 (1.43–2.90)
7	1.83 (1.24–2.69)	1.15 (0.95–1.38)	2.04 (1.43–2.90)
8	1.94 (1.32–2.86)	1.12 (0.93–1.34)	2.04 (1.43–2.90)
9	1.79 (1.19–2.69)	1.12 (0.93–1.34)	2.04 (1.43–2.90)
10	1.82 (1.21–2.75)	1.12 (0.93–1.34)	2.04 (1.43–2.90)
**Combined**	1.83 (1.24–2.69)	1.12 (0.93–1.34)	2.04 (1.43–2.90)

### 3.4 Certainty of Evidence (GRADE Assessment)

Based on the GRADE assessment, the overall certainty of evidence regarding the association between azathioprine use and skin cancer in organ transplant recipients was rated as moderate for NMSC, SCC, and BCC as presented in Table 5. The risk of bias was considered serious due to the presence of a few studies with low quality scores, although the majority of studies were of high quality and carried substantial weight in the meta‐analysis. Some concern was noted regarding inconsistency, as several meta‐analyses showed moderate to high heterogeneity based on the reported Cochran's I^2^ values; however, subgroup analyses and visual inspection suggested that this variability did not meaningfully undermine the overall effect estimates at the direction and magnitude level. No concerns were identified regarding indirectness, imprecision, or publication bias, as all included studies directly assessed the population, exposure, and outcomes of interest, confidence intervals were generally precise, and funnel plot analyses with trim‐and‐fill adjustments minimized the influence of small studies. Therefore, despite the serious risk of bias and some unexplained heterogeneity, the evidence was sufficiently robust to warrant a moderate level of certainty.

**TABLE 5:** GRADE certainty of evidence for the association between azathioprine use and skin cancer in organ transplant recipients.

Outcome	Risk of bias	Inconsistency	Indirectness	Imprecision	Publication bias	GRADE
NMSC–OR/RR/HR	Serious, downgraded one level	Some concern, downgraded by half level	None	None	None	**Moderate**
SCC–OR/RR/HR	Serious, downgraded one level	Some concern, downgraded by half level	None	None	None	**Moderate**
BCC–OR/RR/HR	Serious, downgraded one level	Some concern, downgraded by half level	None	None	None	**Moderate**

*Note:* The results of the GRADE assessment are formatted in bold.

## 4 Discussion

Clinicians should be aware of the high risk of skin cancer development after solid organ transplantation and acquire basic knowledge of its epidemiology, risk factors, diagnosis, preventive strategies, and treatments. OTRs have a higher risk of developing skin cancer, especially SCC [36]. OTRs are 65 to 250 times more likely to develop SCC compared to the general population [37]. Additionally, other types of skin cancer such as BCC, melanoma, and Merkel cell carcinoma are also being reported more frequently in OTRs [5].

As the most common cancer after organ transplantation, skin cancer is a significant cause of illness and death in this population [38]. The standardized incidence ratio (SIR) for SCC ranges from 65 to 250, while the SIR for BCC is 10, resulting in an inversion of the BCC/SCC ratio. BCC is more prevalent in the first years after organ transplantation, with the risk increasing linearly over time. On the other hand, the risk of SCC rises exponentially [39].

There is a dearth of real‐world data regarding durable effectiveness, safety, and clinical outcomes associated with long‐term AZA usage [40]. Previously, one meta‐analysis has been conducted to assess the connection between AZA use and the risk of non‐melanoma skin cancers among OTR. The study found a positive correlation between AZA use and the risk of SCC (estimate effect = 1.56, 95% CI = 1.11 to 2.18). However, the study couldn't find a significant relationship between BCC and the combined risk of SCC and BCC labeled as KC (BCC estimate effect = 0.96, 95% CI = 0.66 to 1.40, KC estimate effect = 0.84, 95% CI = 0.59 to 1.21) recipients [6]. Furthermore, the study was conducted until 2016, and the authors did not include the Web of Science database. Additionally, all types of estimated effects of the included studies, including OR, RR, and HR, were pooled together and not analyzed separately. Therefore, the findings of this meta‐analysis require updating.

The study shows that the use of AZA is a risk factor for NMSC incidence (OR = 1.83, with a 95% CI 1.22 to 2.75). The (RR = 2.09, with a 95% CI of 1.41 to 3.10). (HR = 1.12, with a 95% CI 0.93 to 1.34). In subgroup analysis, the study found a strong relationship between AZA use and the risk of SCC in all estimate effects (OR = 2.67, 95% CI = 1.72 to 4.14; RR = 2.59, 95% CI = 1.46 to 4.59; HR = 1.26, 95% CI = 1.01 to 1.57). The study also found a positive correlation between NMSC and treated with AZA (OR = 1.81, 95% CI = 1.09 to 2.98; RR = 1.50, 95% CI = 1.14 to 1.97). However, the study did not find any significant association between BCC and treated with AZA in any of the estimate effects. Therefore, we can conclude that there is no higher risk of BCC after an immunosuppressive regimen that includes AZA in OTR.

Among the risk factors for the development of SCC after organ transplantation are older age at the time of transplantation, light skin type, high sun exposure, frequent sunburns in childhood, history of skin cancer before transplantation, and rejection periods in the first year of transplantation [41]. According to the results of this meta‐analysis, the use of AZA also increases the risk of skin cancers, so it is important to adjust immunosuppressive regimens after transplantation to prevent adverse reactions in high‐risk patients or those with special conditions.

AZA increases sensitivity to UVA light, which could potentially lead to skin cancer. Additionally, AZA inhibits DNA synthesis in rapidly dividing cells, including those of the immune system. This helps prevent organ rejection in transplant patients but also hinders the immune system's ability to eliminate UV‐damaged cells, potentially allowing them to develop into malignancies [6, 42]. Azathioprine increases the risk of skin cancer in organ transplant recipients by incorporating 6‐thioguanine (6‐TG) into DNA, which sensitizes cells to UVA. UVA exposure then induces DNA and protein damage, strand breaks, and crosslinks, while azathioprine impairs immune surveillance of damaged cells. These combined effects promote genomic instability and elevate the risk of squamous cell carcinoma, highlighting the need for UV protection and regular dermatologic monitoring [43].

The current study has limitations as it did not exhaustively search all electronic databases, potentially excluding relevant studies. Consequently, the studies incorporated into this meta‐analysis might not fully represent the universe of potential research. However, the statistical tests and funnel plots revealed minimal bias stemming from the omission of potential studies in the pooled estimates. This recognition underscores a limitation in our study. Nevertheless, with a substantial subject pool of 21 405 individuals, and a comprehensive subgroup analysis across 6 different subgroups, the present study consistently indicates that AZA is a significant risk factor for the development of SCC. This study conducted a comprehensive subgroup analysis across six different subgroups.

Further high‐quality studies are needed using standard categorization to measure AZA exposure and enable study comparisons. We also strongly recommend that future studies separately examine SCC and BCC risk. Given the elevated risk of SCC in patients receiving azathioprine, clinicians should ensure regular dermatologic surveillance, particularly for high‐risk individuals. While azathioprine remains an effective immunosuppressant, alternative agents such as mycophenolate may be considered in patients at increased risk, balancing efficacy and safety. These strategies aim to translate our findings into actionable clinical guidance for post‐transplant patient management.

## 5 Conclusion

Clinicians should be aware of the high risk of skin cancer development following solid organ transplantation. Our findings indicate that OTRs treated with AZA are at an increased risk for SCC and NMSC. Therefore, it is recommended to prioritize monitoring for skin cancer in OTRs treated with AZA.

## Author Contributions

Conceptualization: A.M.S. and B.E. Methodology: A.M.S. and R.R. Software: S.K. Investigation: A.M.S. Data curation: A.S. and R.R. Writing and original draft preparation: A.S. and R.R. Writing, review and editing: A.M.S. Supervision: B.E. Project administration: S.K. All authors have read and agreed to the published version of the manuscript.

## Funding

This study was financially supported by the Vice Chancellor for Research and Technology, Hamadan University of Medical Sciences, Hamadan, Iran (140311029895).

## Ethics Statement

The Ethics Committee of the Hamadan University of Medical Sciences approved the protocol of this study (IR.UMSHA.REC.1403.818).

## Consent

The authors have nothing to report.

## Conflicts of Interest

The authors declare no conflicts of interest.

## Supporting information


**Data S1:** Preferred Reporting Items for Systematic Reviews and Meta‐Analyses (PRISMA) checklist.

**Data S2:** Exclusion studies with reasons.

## Data Availability

The data that support the findings of this study are available from the corresponding author upon reasonable request.
