# Supplementary material for: Azathioprine Increases the Risk of Non‐Melanoma Skin Cancer Among Organ Transplant Recipients; an Updated Systematic Review and Meta‐Analysis
Source: Cancer Rep (Hoboken). 2026 Feb 10;9(2):e70473. doi: 10.1002/cnr2.70473 (PMC12890441; doi:10.1002/cnr2.70473)
Supplement: Supplementary file 2 — Data S2: Exclusion studies with reasons. [file CNR2-9-e70473-s002.pdf]

Reasons for exclusion of studies:

Estimate of effect (OR/ HR/ RR) was not reported

1. Kuijken I, Bavinck JN. Skin cancer risk associated with immunosuppressive therapy in organ transplant recipients: epidemiology and proposed mechanisms. BioDrugs. 2000;14(5):319-29.

2. Preciado DA, Matas A, Adams GL. Squamous cell carcinoma of the head and neck in solid organ transplant recipients. Head Neck. 2002;24(4):319-25.

3. Herrero JI, España A, Quiroga J, Sangro B, Pardo F, Alvárez-Cienfuegos J, et al. Nonmelanoma skin cancer after liver transplantation. Study of risk factors. Liver Transpl. 2005;11(9):1100-6.

4. Perera GK, Child FJ, Heaton N, O'Grady J, Higgins EM. Skin lesions in adult liver transplant recipients: a study of 100 consecutive patients. Br J Dermatol. 2006;154(5):868-72.

5. Gallagher MP, Kelly PJ, Jardine M, Perkovic V, Cass A, Craig JC, et al. Long-term cancer risk of immunosuppressive regimens after kidney transplantation. J Am Soc Nephrol. 2010;21(5):852-8.

6. Oliveira WRP, Tirico M, Souza AAV, Codarin FR, Silva LLC, Festa Neto C. Skin lesions in organ transplant recipients: a study of 177 consecutive Brazilian patients. Int J Dermatol. 2019;58(4):440-8.

7. Kim YJ, Jung CJ, Park GH, Won CH, Chang SE, Choi JH, et al. Twenty-eight-year incidence and characteristics of post-transplant skin cancers: Comparative analysis of past and recent 10-year experience. J Dermatol. 2020;47(10):1131-40.

8. Shao EX, Betz-Stablein B, Marquat L, Campbell S, Isbel N, Green AC, et al. Higher mycophenolate dosage is associated with an increased risk of squamous cell carcinoma in kidney transplant recipients. Transpl Immunol. 2022;75:101698.

9. Patel GM, Parveen S, Mazumder A. Risk factors and treatment for Non-Melanoma Skin Cancer (N-MSC) in solid organ transplant recipients. Onkologia i Radioterapia. 2024;18(7).

Nonmelanoma skin cancer outcomes were not reported separately (malignancy, lip cancer or melanoma skin cancer)

1. Ondrus D, Pribylincová V, Breza J, Bujdák P, Miklosi M, Reznícek J, et al. The incidence of tumours in renal transplant recipients with long-term immunosuppressive therapy. Int Urol Nephrol. 1999;31(4):417-22.

2. Robbins HA, Clarke CA, Arron ST, Tatalovich Z, Kahn AR, Hernandez BY, et al. Melanoma Risk and Survival among Organ Transplant Recipients. J Invest Dermatol. 2015;135(11):2657-65.

3. Na R, Laaksonen MA, Grulich AE, Meagher NS, McCaughan GW, Keogh AM, et al. High azathioprine dose and lip cancer risk in liver, heart, and lung transplant recipients: A population-based cohort study. J Am Acad Dermatol. 2016;74(6):1144-52.e6.

4. Laprise C, Cahoon EK, Lynch CF, Kahn AR, Copeland G, Gonsalves L, et al. Risk of lip cancer after solid organ transplantation in the United States. Am J Transplant. 2019;19(1):227-37.

5. Nejatifar F, Monfared A, Khosravi M, Lebadi M, Shakiba M, Mokhtari G, et al. Incidence and Risk Factors of Post-renal Transplantation Malignancies in North of Iran, A 20-year Experience. Iran J Kidney Dis. 2020;14(6):439-47.

6. Shaw R, Haque AR, Luu T, O'Connor TE, Hamidi A, Fitzsimons J, et al. Multicenter analysis of immunosuppressive medications on the risk of malignancy following adult solid organ transplantation. Front Oncol. 2023;13:1146002.

7. Wimmer CD, Rentsch M, Crispin A, Illner WD, Arbogast H, Graeb C, et al. The janus face of immunosuppression - de novo malignancy after renal transplantation: the experience of the Transplantation Center Munich. Kidney Int. 2007;71(12):1271-8.

There was no control group

1. Ducroux E, Martin C, Bouwes Bavinck JN, Decullier E, Brocard A, Westhuis-van Elsäcker ME, et al. Risk of Aggressive Skin Cancers After Kidney Retransplantation in Patients With Previous Posttransplant Cutaneous Squamous Cell Carcinomas: A Retrospective Study of 53 Cases. Transplantation. 2017;101(4):e133-e41.

Interventional studies (randomized controlled trials)

1. Eisen HJ, Kobashigawa J, Keogh A, Bourge R, Renlund D, Mentzer R, et al. Three-year results of a randomized, double-blind, controlled trial of mycophenolate mofetil versus azathioprine in cardiac transplant recipients. J Heart Lung Transplant. 2005;24(5):517-25.

2. Gallagher MP, Kelly PJ, Jardine M, Perkovic V, Cass A, Craig JC, et al. Long-term cancer risk of immunosuppressive regimens after kidney transplantation. J Am Soc Nephrol. 2010;21(5):852-8.
